# Immunization Against Poliomyelitis and the Challenges to Worldwide Poliomyelitis Eradication

**DOI:** 10.1093/infdis/jiaa622

**Published:** 2021-09-30

**Authors:** John F Modlin, Ananda S Bandyopadhyay, Roland Sutter

**Affiliations:** 1 Professor of Pediatrics and Medicine (Emeritus), Geisel School of Medicine, Dartmouth College, Hanover, New Hampshire, USA; 2 Retired (formerly Bill & Melinda Gates Foundation), Seattle, Washington, USA; 3 Polio, Bill & Melinda Gates Foundation, Seattle, Washington, USA; 4 Retired (formerly World Health Organization), Geneva, Switzerland

**Keywords:** poliomyelitis, inactivated poliovirus vaccine, oral poliovirus vaccine, polio eradication

## Abstract

Both inactivated poliovirus vaccine (IPV) and oral poliovirus vaccine (OPV) have contributed to the rapid disappearance of paralytic poliomyelitis from developed countries despite possessing different vaccine properties. Due to cost, ease of use, and other properties, the Expanded Programme on Immunization added OPV to the routine infant immunization schedule for low-income countries in 1974, but variable vaccine uptake and impaired immune responses due to poor sanitation limited the impact. Following launch of the Global Polio Eradication Initiative in 1988, poliomyelitis incidence has been reduced by >99% and types 2 and 3 wild polioviruses are now eradicated, but progress against type 1 polioviruses which are now confined to Afghanistan and Pakistan has slowed due to insecurity, poor access, and other problems. A strategic, globally coordinated replacement of trivalent OPV with bivalent 1, 3 OPV in 2016 reduced the incidence of vaccine-associated paralytic poliomyelitis (VAPP) but allowed the escape of type 2 vaccine–derived polioviruses (VDPV2) in areas with low immunization rates and use of monovalent OPV2 in response seeded new VDPV2 outbreaks and reestablishment of type 2 endemicity. A novel, more genetically stable type 2 OPV vaccine is undergoing clinical evaluation and may soon be deployed prevent or reduce VDPV2 emergences.

The conquest of polio is widely acknowledged to be one of the great scientific and medical achievements of the 20th century. The story is well known; periodic poliomyelitis epidemics first appeared in Scandinavia, Western Europe, and the United States in the late 1800s and steadily grew in frequency and magnitude until the 1950s and the early 1960s when inactivated poliovirus vaccine (IPV) and, later, live attenuated oral poliovirus vaccine (OPV) were introduced, leading to immediate and dramatic control of poliomyelitis in the United States and other industrialized countries [1]. In the United States, polio incidence fell from 13.9 cases per 100 000 in 1954 to <0.5 cases per 100 000 in 1965, endemic transmission ceased by 1970, and the last case of domestically acquired poliomyelitis was reported in 1979. Thereafter, only rare imported cases or cases caused by the live attenuated OPV (ie, vaccine-associated paralytic poliomyelitis [VAPP]) were observed, and today imported poliomyelitis has all but vanished with global control of polio and adoption of IPV-only schedules in many countries.

However, in low-income countries with crowding and poor sanitation, recognition of the impact of poliomyelitis was long obscured by universal infection at an early age and the dogma that epidemic poliomyelitis was a disease of wealthier nations because exposure at older ages in countries with better hygiene increased the risk of paralytic disease. This misconception was rectified when many community- and school-based lameness surveys documented that approximately 1:100 to 1:200 children in poor countries suffered the paralytic consequences of poliomyelitis, rivaling the incidence rates observed in peak epidemic years in developed countries [2]. These observations supported the World Health Organization (WHO) decision to include OPV in the Expanded Programme on Immunization (EPI) beginning in 1974 [3].

## POLIO VACCINES

While the relative merits of IPV and OPV have been debated since their introduction, each vaccine has contributed to the control and, optimistically, to the eventual eradication of poliomyelitis (Table 1).

### Inactivated Poliovirus Vaccine

IPV is produced according to methods originally developed by Jonas Salk and Julius Youngner with the use of formalin inactivation of 3 well-characterized poliovirus strains originally isolated from poliomyelitis patients, each representing a single serotype. The first IPV vaccines tested in the Francis Field Trial collectively protected approximately 70% of children from paralytic poliomyelitis following 3 doses given within a 5-week period [4]. Improvements in production technology in the 1970s, including the use of microcarrier cultivation to increase cell density, led to the introduction of “enhanced potency” formulations meeting a universal standard of 40, 8, and 32 D antigen units, for types 1, 2 and 3, respectively, per dose. The immune response to IPV depends on the number of doses, the interval between doses, and the presence of maternally derived antibody, which impairs immune responses in infants up to 6 months of age [5–7]. Detectable serum neutralizing antibody protects against disease, and a ≥4-fold rise in neutralizing antibody titer is widely accepted as the standard defining seroconversion [8]. When administered according to common recommendations, a 3-dose infant immunization schedule results in seroconversion rates of 85%–100% against each serotype [9]. When used as a single IPV dose at 14 weeks of age to supplement bivalent OPV (bOPV) type 1 and 3 vaccine according to current WHO recommendations, type 2 seroconversion rates are approximately 65%–70% [10]. IPV produced from attenuated Sabin OPV viruses is now licensed in Japan and China and will soon be licensed in other countries, starting with Korea [11].

### Oral Poliovirus Vaccine

The Sabin live attenuated OPV vaccine strains were created by passage of polioviruses in cultured primate cells and, for type 2, also in chimpanzees, followed by selection of mutants with low virulence for primates [12]. Successful field trials were carried out from 1955 to 1959 and OPV was introduced for routine use between 1961 and 1962 as sequentially administered monovalent vaccines. Trivalent OPV (tOPV) became available in 1964 and was rapidly adopted for infant immunization in all but a few countries due to superior immunogenicity compared with the IPV vaccines then available, lower cost, and ease of administration. Although rare cases of VAPP were recognized shortly after the introduction of OPV [13], tOPV remained the vaccine of choice in most countries until the late 1990s and early 2000s when even a small number of VAPP cases became untenable in the absence of cases caused by wild-type polioviruses (WPVs) and the introduction of IPV in combination with diphtheria, tetanus, and pertussis (DTP) and other infant vaccines in middle- and high-income countries [14].

The low cost, ease of administration, spread of vaccine virus to unimmunized, susceptible persons, and induction of gastrointestinal immunity make OPV the logical choice for continued use in low-income countries. For many years, tOPV was delivered for routine immunization through the EPI and via campaigns by the Global Polio Eradication Initiative (GPEI). After 2005, monovalent vaccines for type 1 (mOPV1) and type 3 (mOPV3), and later type 1 and 3 bOPV vaccine, were deployed in supplementary immunization campaigns in the remaining polio-endemic countries to overcome the problem of intertypic interference from the type 2 OPV in the trivalent vaccine and enhance seroconversion to types 1 and 3 [15]–17]. The use of these vaccines in campaigns was followed by a globally coordinated “switch” from tOPV to bOPV for routine immunization in 2016 based on improving immune responses to poliovirus types 1 and 3 and preventing new VAPP cases from OPV2 [18]. Currently, tOPV is no longer available, and monovalent mOPV type 2 can be released only for control of vaccine–derived poliovirus type 2 (VDPV2) outbreaks by the WHO Director-General.

A large study in India found that 2 bOPV doses at birth and 28 days of age induced seroconversion rates of 86% and 74% to type 1 and type 3 polioviruses, respectively, superior to respective rates of 63% and 52% observed with a comparator tOPV vaccine and noninferior to monovalent OPV1 and OPV3 vaccines [19]. Similarly, mOPV2 vaccine has high immunogenicity in infants, with seroconversion rates approaching 90% for 1 dose [20], [21]. In addition to intertypic interference, the immune response to OPV is further reduced by chronic malnutrition, enteric enteropathy, diarrheal disease, and concurrent infections with other enteroviruses [22–24]. As a consequence, a larger number of OPV doses is required to achieve protective immunity and reduce poliovirus transmission among children living in adverse environments [25, 26].

VAPP is a rare, but serious complication of OPV use observed in OPV recipients and their contacts that is clinically indistinguishable from naturally occurring poliomyelitis. Prior to 2014, the global incidence of VAPP was estimated to be 2–4 cases/10^6^ birth cohort (250–500 cases) per year in tOPV-using countries [27],[28]. Since the removal of OPV2 and addition of IPV to the EPI schedule, the estimated VAPP incidence has fallen below 2 cases/10^6^ (WHO Polio, unpublished data). The risk of VAPP has been a dominant factor in global polio immunization policy, leading to the decision to discontinue OPV2 use in 2016 and to the plan to cease all OPV use in order to eradicate polio worldwide [27].

## THE GLOBAL POLIO ERADICATION INITIATIVE

The success of the smallpox eradication program in the 1970s led to the establishment of the EPI in 1974 when the public health community increasingly sought to harvest the full potential of vaccines. Rotary International had targeted poliomyelitis prevention as a priority and began to fund poliovirus vaccines for some of the poorest countries. In 1985, the Pan American Health Organization (PAHO) resolved to eliminate poliomyelitis from the Western Hemisphere. Rapid progress toward this goal encouraged the global public health community to consider whether poliomyelitis might be a candidate for a global eradication target. At that time, many policy makers had personal experience with children developing this crippling disease and were committed to prevent this illness, and technical reviews rated polio as the top candidate on the list of potential eradicable diseases.

These developments led to a 1988 resolution by the World Health Assembly, the governing body of WHO, to eradicate poliomyelitis by the year 2000 [29]. GPEI, composed of Rotary International, the United States Centers for Disease Control and Prevention, the United Nations Children’s Fund, and the WHO, was created to lead this effort. The Bill & Melinda Gates Foundation and Gavi, the Vaccine Alliance subsequently joined as partners. Full engagement by national ministries of health, nongovernmental organizations, donor agencies, and philanthropic organizations has been critical to the success of the program.

### Progress

**Figure 1.**
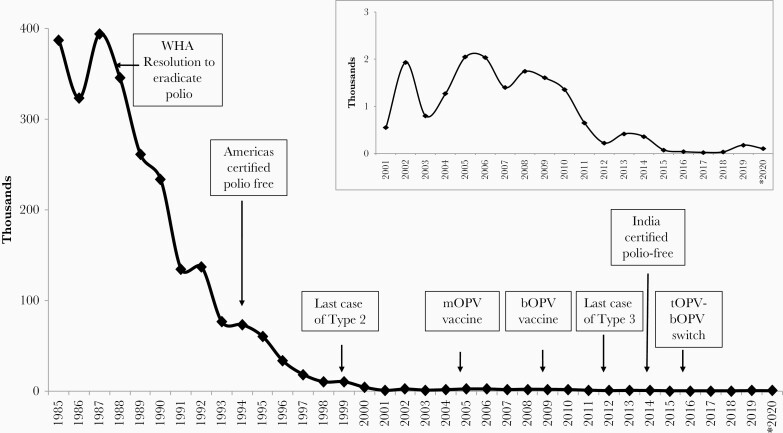
Reported global wild type poliomyelitis cases, 1985–2020. World Health Organization data. *As of 25 August 2020. Abbreviations: bOPV, bivalent oral poliovirus vaccine; mOPV, monovalent oral poliovirus vaccine; tOPV, trivalent OPV; WHA, World Health Assembly; WHO, World Health Organization.

When the GPEI was established in 1988, >125 countries had an estimated poliomyelitis burden that totaled >350 000 cases annually. Implementation of core strategies initially developed by PAHO and adapted to polio-endemic countries in Asia and Africa, including acute flaccid paralysis surveillance, supplementary immunization campaigns, and support for routine infant tOPV immunization, led to a rapid decrease in global polio incidence, first in countries with better-developed health systems, including the Americas where the last indigenous WPV case was detected in 1991; the WHO Western Pacific Region, including China, where the last case was observed in 1997; and in Europe, where the same milestone was reached in 1999 (Figure 1) [30–32].

By the mid-1990s, eradication efforts were fully operational in all polio-endemic countries including India, where >50% of the world`s poliomyelitis cases were reported, and in sub-Saharan Africa, where a football (soccer)–themed initiative, “Kick Polio Out of Africa,” was launched in 1997 [33]. By 2000, although the original goal of eradication was not achieved, the number of countries reporting poliomyelitis had declined to 20 and the number of cases had decreased by >99%. Furthermore, the last indigenous wild poliovirus type 2 (WPV2) case was detected in northern India in 1999 [34].

Rapid progress against type 1 and 3 polioviruses continued into the new millennium with a reduction of the number poliomyelitis cases from WPVs to a few hundred by 2010, all of which were confined to just 4 endemic countries (Afghanistan, India, Nigeria, and Pakistan). Eradication activities now focused increasingly on countries with suboptimal health systems in Asia, the Middle East, and Africa, where frequent importation of WPVs from the endemic countries, primarily type 1, created special challenges, as did recognition that the immunogenicity of tOPV was low in tropical developing countries, especially in the Indian subcontinent [25]. To improve the immune responses to OPV vaccines delivered in large-scale supplementary immunization campaigns, mOPV1 and mOPV3 were introduced in 2005, and bOPV containing types 1 and 3 was licensed and used for the first time in 2009 [35]. The introduction of the mOPV vaccines and bOPV is credited for eliminating the final chains of WPV transmission in India, where the last case of indigenous type 1 wild poliovirus (WPV1) was detected in January 2011, leading to certification of the entire WHO Region of South Asia as free of polio in 2014 by an independent commission [36]. Wild type 3 poliovirus (WPV3) circulation was last reported in northern Nigeria in 2012, leading to certification of global WPV3 eradication in 2019. In 2020, the African Region of WHO was certified as free of wild poliovirus following the last observed case in Nigeria in 2016.

During the past decade, transmission of remaining WPV1 has been limited to Afghanistan and Pakistan, and the global incidence fell to an all-time nadir of <40 cases per year between 2016 and 2018 (Figure 1). However, poliovirus control efforts in these countries have since stagnated due to limited access to vaccination due to unrest, civil war, or insurgency; the insufficient quality of polio eradication operations, especially low vaccine coverage; and now compromises imposed by the coronavirus pandemic. Insecurity, access problems, and misinformation all need to be addressed to achieve elimination of the last chains of WPV1 transmission in these countries. As of mid-2020, WPV1 circulation remains confined to Afghanistan and Pakistan, with 33, 176, and 146 reported cases in 2018, 2019, and 2020, respectively (WHO data as of 12 January 2021).

### Vaccine-Derived Polioviruses and the tOPV-to-bOPV Switch

Challenges not anticipated at time of the World Health Assembly resolution in 1988 have slowed progress and required the commitment of substantial resources to combat, most notably, the emergence and spread of circulating vaccine-derived polioviruses (cVDPVs). By the late 1990s, advances in viral genetic sequencing technology led to the discovery of a circulating type 1 VDPV as the cause of an outbreak of poliomyelitis on Hispaniola, demonstrating for the first time that the attenuated Sabin poliovirus strains in the OPV could mutate, assume the transmissibility and neurovirulence characteristics of WPV, and initiate chains of virus transmission resulting in epidemic transmission [37]. It soon became apparent that continuous use of Sabin strains was incompatible with complete poliomyelitis eradication, which led to a decade-long effort to characterize the risk of cVDPV emergence including modeling, attempts to close important research gaps, and extensive consultation with experts in the global health community. The resulting strategic plan called for the sequential removal of Sabin strains from the OPV, starting with Sabin type 2, and the introduction of 1 or more doses of IPV in the EPI schedule to provide an immunity base against paralytic disease and mitigate the risk of cVDPV emergence [18]. With the approval of the World Health Assembly, the governing body of WHO, the plan was implemented in April 2016 with a coordinated “switch” from tOPV to bOPV in nearly 125 countries [38]. However, the switch was accompanied by delays in introduction of IPV due to supply constraints that affected 43 countries but which were mostly resolved by late 2019.

### Emergence and Expansion of cVDPVs

**Figure 2.**
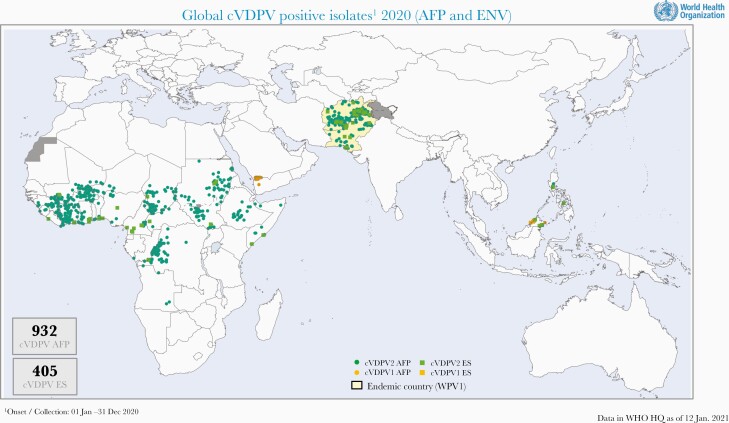
Global circulating vaccine-derived poliomyelitis isolates and response campaigns, 1 January–31 December 2020. Courtesy Ondrej Mach and Ajay Kumar Goel, World Health Organization. Abbreviations: AFP, acute flaccid paralysis; bOPV, bivalent oral poliovirus vaccine; cVDPV, circulating vaccine-derived poliomyelitis; ES, environmental surveillanve; WHO, World Health Organization.

The withdrawal of Sabin 2, together with the addition to introduction of IPV in the routine immunization schedule, reduced the VAPP burden by about 60%, preventing between 150 and 300 cases annually worldwide. Unfortunately, continuous circulation of cVDPVs that carried over from the preswitch period and the growing type 2 immunity gap that developed after the switch from trivalent OPV to bOPV facilitated more VDPV2 outbreaks than anticipated from contemporary models, and use of mOPV2 vaccine for outbreak control seeded more emergences [39]. Between 2016 and September 2020, cVDPV2 outbreaks spread to 24 countries, mostly in sub-Saharan Africa but also areas of Pakistan, Afghanistan and China, the Philippines, and Malaysia, requiring multiple rounds of mOPV2 or bOPV in response (Figure 2) [40]. In 2019, more children were paralyzed in 2020 by cVDPV2 (n = 918) than by WPV (n = 140) [41]. As long as control activities do not achieve an adequate level of population immunity in affected areas, new outbreaks will continue to emerge in areas receiving multiple rounds with mOPV2, which puts the world at high risk of reestablishing poliovirus type 2 endemic transmission.

### Chronic Vaccine-Derived Poliovirus Infection in Congenital Immunodeficiency Disorders

Another challenge to achieving and maintaining long-term polio eradication is posed by rare persons with inherited immunodeficiency disorders who may excrete VDPV for very long periods and represent a risk to seed new outbreaks where transmission has otherwise ceased [42]. A program led by the Task Force for Global Health to develop antiviral drugs to clear these viral infections was initiated more than a decade ago. Pocapavir, a capsid inhibitor developed by the Task Force for Global Health consortium, has demonstrated efficacy in a human OPV challenge trial, and a second agent V-7404, a 3C protease inhibitor, is currently in phase 2 clinical development [43]. Early evidence suggests that effective treatment may require a combination of drugs to reduce development of resistance.

## NEW POLIO VACCINES

The challenge of achieving and sustaining eradication has created a clear need for new poliovirus vaccines. New vaccines that build on existing platforms for IPV are being introduced, and considerable progress is being made toward new IPV and new OPV vaccines that are adjuvanted, genetically modified, or rely on new production technologies, to both address current and future needs (Table 2).

### Inactivated Poliovirus Vaccines

As the risk of poliovirus transmission wanes, OPV routine childhood immunization will be replaced country by country with IPV to prevent the emergence of VDPVs. The WHO Strategic Advisory Group of Experts on Immunization has recommended that global use of IPV continue for a minimum of 10 years after OPV cessation and indefinitely in countries that host essential poliovirus facilities, including IPV manufacturers, in order to mitigate the risks of recurrent poliovirus outbreaks seeded by undetected cVDPVs, immune deficient poliovirus excretors, or environmental exposure following accidental release from a manufacturing facility or laboratory [44]. This creates a demand for greater IPV supplies at a cost much lower than the current price in order to make IPV accessible to all low- and moderate-income countries. Various strategies are being developed to meet this need, including antigen sparing and new vaccine production technologies.

One practical approach to IPV antigen sparing is use of fractional doses. Two 0.1-mL doses (one-fifth the standard volume) delivered intradermally results in seroconversion rates that are equal to or exceed those from a single full dose administered intramuscularly, while achieving 60% dose sparing [45]. In response to IPV supply constraints, intradermal administration for routine infant immunization has been adopted in India and other South Asian countries. A recent study in Cuba suggests that similar dose sparing can be achieved with fractional doses given intramuscularly, which would abrogate the need for the special equipment and training associated with intradermal administration [46].

Antigen sparing can also be achieved by inclusion of an adjuvant, which lowers the antigen contect required to induce an immune response comparable to unadjuvanted IPV. An alum adjuvanted IPV containing one-tenth the antigen content of conventional IPV was recently licensed in Denmark and will soon be available to the global market [47].

A potent adjuvant derived from *Escherichia coli*, double-mutant labile toxin (dmLT), not only enhances serum neutralizing antibody responses by >5-fold when administered either intradermally or intramuscularly in a murine model, but uniquely induces antipoliovirus immunoglobulin A secretion in the gut, raising the possibility that, unlike conventional IPV, an adjuvanted inactivated vaccine might reduce poliovirus transmission similar to that of OPV [48, 49]. The first-in-human studies with dmLT-IPV are currently in progress.

Considerable progress has been made toward reduction of long-term risks associated with IPV manufacturing by adoption of seed strains that are less virulent than the wild-type viruses used for conventional IPV production. New Sabin strain vaccines (sIPVs) carry lower biosafety risks in addition to contributing needed supplies to the global market. The sIPV was first licensed in Japan in 2012 in combination with DTP vaccine, and 2 standalone sIPV vaccines have since been approved in China [50, 51]. Additional manufacturers, some in developing countries, plan to produce sIPV for the global market as participants in a WHO-sponsored technology transfer initiative, and recently standardized reagents have been approved by WHO to assist in sIPV development, manufacturing, and regulatory practice [52].

Because Sabin vaccine stains still carry a risk of reversion to neurovirulence, evaluation of IPVs produced with “further attenuated” S19 Sabin derivatives that do not replicate in humans is now in progress [53, 54]. In addition, progress has been made in development of IPV with genetically engineered virus-like particles, which would avoid virus cultivation altogether [55].

### Oral Poliovirus Vaccines

A project to develop Sabin strain derivatives that are less susceptible to reversion to the wild-type phenotype was initiated by the Bill & Melinda Gates Foundation nearly a decade ago and has now taken on new urgency with the emergence of multiple VDPV2 outbreaks since 2017. Candidate novel oral poliovirus vaccine (nOPV) strains have been generated for all 3 types, with development of nOPV2 given priority. The modifications to the Sabin genome in the nOPV2 vaccine now in clinical development are designed to stabilize key attenuating mutations in the 5′-nontranslated region, suppress recombination with other species C enteroviruses, and limit viral adaptability [56]. Early-phase clinical testing indicates that nOPV2 is safe, immunogenic, and genetically stable on passage through the gastrointestinal tract, but these results require confirmation through field use and larger trials [57]. The nOPV2 is currently being reviewed under a WHO Emergency Use Listing for early application in the field in regions affected by VDPV2 outbreaks [58]. Ultimately, availability of nOPVs of each serotype will strengthen the prospects of successful polio eradication.

## CONCLUSIONS

Although much progress has been achieved in reducing poliomyelitis incidence and extent of virus transmission since 1988, the eradication target remains elusive and the outlook mixed. Two of the 3 WPV serotypes are certified to be eradicated, and WPV1 transmission appears to have ceased in the WHO African Region, which was recently certified to be free of WPV transmission. The relative rebound of WPV1 cases in Afghanistan and Pakistan since 2018 should not detract from the progress to date, but signals that further intensified efforts will be required to complete interruption of transmission of the only remaining wild-type polioviruses. The success against WPVs must now be balanced against the emergence of cVDPV2 outbreaks in Africa and Asia, which are abetted by use of mOPV2, the only vaccine available to control outbreaks, and the competing global demands imposed by the coronavirus pandemic.

Despite the extensive and sustained progress, the GPEI has not achieved eradication. Enormous efforts will be needed to reach the eradication finish line that include new commitment to interrupt WPV1 transmission in Asia and to interrupt transmission of type 2 polioviruses originating from OPVs, in addition to new strategies that include surveillance innovation and development and deployment of new polio vaccines designed to support and sustain the GPEI eradication goals [59, 60].

**Table 1.** Current Vaccines and Key Issues Relevant to the Endgame of Polio Eradication

Endgame Issues	IPV (Salk)	OPV (Sabin)
Immunologic		
Primary intestinal mucosal immunogenicity	Minimal	Present
Operational		
SIA use for outbreak response	Difficult	Suitable
Production risks for containment failure	High	Low
Cost	High	Low
Adverse events		
Risk of VAPP	None	Present
Risk of VDPVs	None	Present

Abbreviations: IPV, inactivated poliovirus vaccine; OPV, oral poliovirus vaccine; SIA, supplementary immunization activity; VAPP, vaccine-associated paralytic poliomyelitis; VDPV, vaccine-derived poliovirus.

**Table 2.** New Polio Vaccines

Vaccine Type	Attribute	Examples	Status
Parenteral, inactivated	Dose sparing by fractional dose administration	Intradermal	Adopted in several countries
		Intramuscular	Clinical studies in progress
	Dose sparing by adjuvantation	Alum (AJ Vaccines)	Licensed 2019, WHO prequalified 2020
	Adjuvanted for mucosal immunity	dmLT-IPV	In clinical trial
	Safer production	Sabin IPV	Licensed in Japan, China. WHO prequalified 2020 Other vaccines in advanced clinical development
		S19 IPV	In clinical development
		Virus-like particles	Preclinical
Oral, live attenuated	Enhanced genetic stability	nOPV vaccine candidates	nOPV granted emergency use authorization by WHO; nOPV1 and nOPV3 in preclinical development

Abbreviations: dmLT, double-mutant labile toxin; IPV, inactivated poliovirus vaccine; nOPV, novel oral poliovirus vaccine; OPV, oral poliovirus vaccine; WHO, World Health Organization.

## Supplementary Material

jiaa622_suppl_Supplementary-Material

## Notes

***Supplement sponsorship.*** This supplement is sponsored by the Bill and Melinda Gates Foundation.

***Potential conflicts of interest.*** A. S. B. is employed by the Bill & Melinda Gates Foundation. J. F. M. is a former employee of the Bill & Melinda Gates Foundation. R. S. is a former employee of the World Health Organization.

## Supplementary Data

Supplementary materials are available at *The Journal of Infectious Diseases* online. Consisting of data provided by the authors to benefit the reader, the posted materials are not copyedited and are the sole responsibility of the authors, so questions or comments should be addressed to the corresponding author.See full reference list in the online Supplemental Materials.
