# Supplementary material for: Immunization Against Poliomyelitis and the Challenges to Worldwide Poliomyelitis Eradication
Source: J Infect Dis. 2021 Sep 30;224(Suppl 4):S398–404. doi: 10.1093/infdis/jiaa622 (PMC8482017; doi:10.1093/infdis/jiaa622)
Supplement: jiaa622_suppl_Supplementary-Material [file jiaa622_suppl_supplementary-material.docx]

References

1. Paul JR. History of Poliomyelitis. New Haven: Yale University Press, **1971**.

2. Bernier RH. Some observations on poliomyelitis lameness surveys. Rev Infect Dis **1984**; 6:S371-S5.

3. World Health A. The Expanded Programme on Immunization: the 1974 Resolution by the World Health Assembly. Assignment Child **1985**; 69-72:87-8.

4. Monto AS. Francis field trial of inactivated poliomyelitis vaccine: background and lessons for today. Epidemiological Reviews **1999**; 21:7-23.

5. Sormunen H, Stenvik M, Eskola J, Hovi T. Age- and dose-interval-dependent antibody responses to inactivated poliovirus vaccine. J Med Virol **2001**; 63:305-10.

6. Dayan GH, Thorley M, Yamamura Y, et al. Serologic response to inactivated poliovirus vaccine: a randomized clinical trial comparing 2 vaccination schedules in Puerto Rico. J Infect Dis **2007**; 195:12-20.

7. Salk J, Cohen H, Fillastre C, et al. Killed poliovirus antigen titration in humans. Develop Biol Stand **1978**; 41:119-32.

8. Sutter RW, Pallansch MA, Sawyer LA, Cochi SL, Hadler SC. Defining surrogate serologic tests with respect to predicting protective vaccine efficacy: poliovirus vaccination. Ann N Y Acad Sci **1995**; 754:289-99.

9. Vidor E, Meschievitz C, Plotkin S. Fifteen years of experience of Vero-produced enhanced potency inactivated poliovirus vaccine. Ped Infect Dis J **1997**; 16:312-22.

10. Bandyopadhyay AS, Modlin JF, Wenger J, Gast C. Immunogenicity of New Primary Immunization Schedules With Inactivated Poliovirus Vaccine and Bivalent Oral Polio Vaccine for the Polio Endgame: A Review. Clin Infect Dis **2018**; 67:S35-S41.

11. Sutter RW, Okayasu H, Kieny MP. Next Generation Inactivated Poliovirus Vaccine: The Future Has Arrived. Clin Infect Dis **2017**; 64:1326-7.

12. Sabin AB. Oral polio vaccine: History of its development and use and current challenge to eliminate poliomyelitis from the world. J Infect Dis **1985**; 151:420-36.

13. Henderson DA, Witte JJ, Morris L, Langmuir AD. Paralytic disease associated with oral poliovaccines. J Am Med Assn **1964**; 190:153-60.

14. Alexander LN, Seward JH, Santibanez TA, et al. Vaccine policy changes and epidemiology of poliomyelitis in the United States. J Am Med Assn **2004**; 292:1696-701.

15. Patriarca PA, Wright PF, John TJ. Factors affecting the immunogenicity of oral poliovirus vaccine in developing countries. Rev Infect Dis **1991**; 13:926-39.

16. Jenkins HE, Aylward RB, Gasasira A, et al. Effectiveness of immunization against paralytic poliomyelitis in Nigeria. N Engl J Med **2008**; 359:1666-74.

17. Waggie Z, Geldenhuys H, Sutter RW, et al. Randomized trial of type 1 and type 3 oral monovalent poliovirus vaccines in newborns in Africa. J Infect Dis **2012**; 205:228-36.

18. World Health Organization. Polio Eradication and Endgame Strategic Plan 2013–2018. Geneva: World Health Organization, **2013**.

19. Sutter RW, John TJ, Jain H, et al. Immunogenicity of bivalent types 1 and 3 oral poliovirus vaccine: a randomised, double-blind, controlled trial. Lancet **2010**; 376:1682-8.

20. Caceres VM, Sutter RW. Sabin monovalent oral polio vaccines: review of past experiences and their potential use after polio eradication. Clin Infect Dis **2001**; 33:531-41.

21. Zaman K, Estivariz CF, Morales M, et al. Immunogenicity of type 2 monovalent oral and inactivated poliovirus vaccines for type 2 poliovirus outbreak response: an open-label, randomised controlled trial. Lancet Infect Dis **2018**; 18:657-65.

22. Posey DL, Linkins RW, Couto Oliveria MJ, Monteiro D, Patriarca PA. The effect of diarrhea on oral poliovirus vaccine failure in Brazil. J Infect Dis **1997**; 175:S258-S63.

23. Parker EP, Kampmann B, Kang G, Grassly NC. Influence of enteric infections on response to oral poliovirus vaccine: a systematic review and meta-analysis. J Infect Dis **2014**; 210:853-64.

24. Gilmartin AA, Petri WA, Jr. Exploring the role of environmental enteropathy in malnutrition, infant development and oral vaccine response. Philos Trans R Soc Lond B Biol Sci **2015**; 370.

25. Grassly NC, Fraser C, Wenger J, et al. New strategies for the elimination of polio from India. Science **2006**; 314:1150-3.

26. Voorman A, Hoff NA, Doshi RH, et al. Polio immunity and the impact of mass immunization campaigns in the Democratic Republic of the Congo. Vaccine **2017**; 35:5693-9.

27. World Health Organization. Risk assessment: frequency and burden of VAPP, cVDPV and iVDPV. Report of the interim meeting of the Technical Consultative Group (TCG) on the Global Eradication of Poliomyelitis. Geneva, **2002**.

28. Platt LR, Estivariz CF, Sutter RW. Vaccine-associated paralytic poliomyelitis: a review of the epidemiology and estimation of the global burden. J Infect Dis **2014**; 210 Suppl 1:S380-9.

29. World Health Organization. Global eradication of poliomyelitis by the year 2000. Week Epidemiol Rec **1988**; 63:161-2.

30. Centers for Disease Control and Prevention. Certification of poliomyelitis eradication –Western Pacific Region, October 2000. Morb Mort Wk Rep **2000**; 50:1-5.

31. Centers for Disease Control and Prevention. Progress towards global eradication of poliomyelitis, 2002. Morb Mort Wk Rep **2003**; 52:366-9.

32. Anon. The WHO European Region declared free of polio. Euro surveillance : bulletin Europeen sur les maladies transmissibles = European communicable disease bulletin **2002**; 7:76-7.

33. Anon. Kick polio out of Africa. World Health **1997**; 50:15.

34. Bahl S, Bhatnagar P, Sutter RW, Roesel S, Zaffran M. Global Polio Eradication - Way Ahead. Indian J Pediatr **2018**; 85:124-31.

35. Garon J, Seib K, Orenstein WA, et al. Polio endgame: the global switch from tOPV to bOPV. Expert Rev Vaccines **2016**; 15:693-708.

36. Bahl S, Kumar R, Menabde N, et al. Polio-free certification and lessons learned--South-East Asia region, March 2014. MMWR Morb Mortal Wkly Rep **2014**; 63:941-6.

37. Kew O, Morris-Glasgow V, Landaverde M, et al. Outbreak of poliomyelitis in Hispaniola associated with circulating type 1 vaccine-derived poliovirus. Science **2002**; 296:356-9.

38. Patel M, Zipursky S, Orenstein W, Garon J, Zaffran M. Polio endgame: the global introduction of inactivated polio vaccine. Exp Rev Vaccines **2015**:1-14.

39. Blake IM, Pons-Salort M, Molodecky NA, et al. Type 2 Poliovirus Detection after Global Withdrawal of Trivalent Oral Vaccine. N Engl J Med **2018**; 379:834-45.

40. Macklin GR, O'Reilly KM, Grassly NC, et al. Evolving epidemiology of poliovirus serotype 2 following withdrawal of the serotype 2 oral poliovirus vaccine. Science **2020**; 368:401-5.

41. World Health Organization GPEI. Wild Poliovirus Weekly Update. Available at: <http://www.polioeradication.org/Dataandmonitoring/Poliothisweek.aspx>. Accessed 7/2 2019.

42. Macklin G, Liao Y, Takane M, et al. Prolonged Excretion of Poliovirus among Individuals with Primary Immunodeficiency Disorder: An Analysis of the World Health Organization Registry. Front Immunol **2017**; 8:1103.

43. McKinlay MA, Collett MS, Hincks JR, et al. Progress in the development of poliovirus antiviral agents and their essential role in reducing risks that threaten eradication. J Infect Dis **2014**; 210 Suppl 1:S447-53.

44. World Health Organization. Polio eradication. Week Epidemiol Rec **2017**; 92:308-10.

45. Okayasu H, Sein C, Chang Blanc D, et al. Intradermal Administration of Fractional Doses of Inactivated Poliovirus Vaccine: A Dose-Sparing Option for Polio Immunization. J Infect Dis **2017**; 216:S161-S7.

46. Resik S, Mach O, Tejeda A, et al. Immunogenicity of Intramuscular Fractional Dose of Inactivated Poliovirus Vaccine. J Infect Dis **2020**; 221:895-901.

47. Rivera L, Pedersen RS, Pena L, et al. Immunogenicity and safety of three aluminium hydroxide adjuvanted vaccines with reduced doses of inactivated polio vaccine (IPV-Al) compared with standard IPV in young infants in the Dominican Republic: a phase 2, non-inferiority, observer-blinded, randomised, and controlled dose investigation trial. Lancet Infect Dis **2017**; 17:745-53.

48. Norton EB, Bauer DL, Weldon WC, Oberste MS, Lawson LB, Clements JD. The novel adjuvant dmLT promotes dose sparing, mucosal immunity and longevity of antibody responses to the inactivated polio vaccine in a murine model. Vaccine **2015**; 33:1909-15.

49. Clements JD, Freytag LC. Parenteral Vaccination Can Be an Effective Means of Inducing Protective Mucosal Responses. Clin Vaccine Immunol **2016**; 23:438-41.

50. Okada K, Miyazaki C, Kino Y, Ozaki T, Hirose M, Ueda K. Phase II and III Clinical Studies of Diphtheria-Tetanus-Acellular Pertussis Vaccine Containing Inactivated Polio Vaccine Derived from Sabin Strains (DTaP-sIPV). J Infect Dis **2013**; 208:275-83.

51. Okayasu H, Sein C, Hamidi A, Bakker WAM, Sutter RW. Development of inactivated poliovirus vaccine from Sabin strains: A progress report. Biologicals **2016**; 44:581-7.

52. Crawt L, Atkinson E, Tedcastle A, et al. Differences in Antigenic Structure of Inactivated Polio Vaccines Made From Sabin Live-Attenuated and Wild-Type Poliovirus Strains: Impact on Vaccine Potency Assays. J Infect Dis **2020**; 221:544-52.

53. Knowlson S, Burlison J, Giles E, Fox H, Macadam AJ, Minor PD. New Strains Intended for the Production of Inactivated Polio Vaccine at Low-Containment After Eradication. PLoS Pathog **2015**; 11:e1005316.

54. Biological E L. Inactivated Polio Vaccine. Available at: <http://www.biologicale.com/Vaccines_Biologics/pipeline.html>. Accessed 6/12/2020 2020.

55. Fox H, Knowlson S, Minor PD, Macadam AJ. Genetically Thermo-Stabilised, Immunogenic Poliovirus Empty Capsids; a Strategy for Non-replicating Vaccines. PLoS Pathology **2017**; 13:e1006117.

56. Yeh M-T, Bujaki E, Dolan PT, et al. Engineering the live-attenuated polio vaccine to prevent reversion to virulence. Cell Host & Microbe **2020**; 27:1-16.

57. Van Damme P, De Coster I, Bandyopadhyay AS, et al. The safety and immunogenicity of two novel live attenuated monovalent (serotype 2) oral poliovirus vaccines in healthy adults: a double-blind, single-centre phase 1 study. Lancet **2019**; 394:148-58.

58. Bandyopadhyay AS. Clinical data from novel type-2 oral polio vaccine trials and plan for emergency use listing. In: Meeting of the Strategic Advisory Group of Experts (SAGE) on Immunization, March – April 2020 (Geneva). World Health Organization.

59. World Health Organization. The Polio Endgame Strategy 2019-2023: Eradication, Integration, Containment and Certification. Vol. 2020. Geneva: World Health Organization, **2020**.

60. Bandyopadhyay AS, Macklin GR. Final frontiers of the polio eradication endgame. Curr Opin Infect Dis **2020**.
